# From Oxidative Stress to Inflammation in the Posterior Ocular Diseases: Diagnosis and Treatment

**DOI:** 10.3390/pharmaceutics13091376

**Published:** 2021-08-31

**Authors:** Azza Dammak, Fernando Huete-Toral, Carlos Carpena-Torres, Alba Martin-Gil, Cristina Pastrana, Gonzalo Carracedo

**Affiliations:** 1 Ocupharm Group Research, Faculty of Optic and Optometry, University Complutense of Madrid, C/Arcos del Jalon 118, 28037 Madrid, Spain; Azzadamm@ucm.es (A.D.); fhueteto@ucm.es (F.H.-T.); ccarpena@ucm.es (C.C.-T.); amarting@ucm.es (A.M.-G.); crispast@ucm.es (C.P.); 2 Department of Optometry and Vsiion, Faculty of Optic and Optometry, University Complutense of Madrid, C/Arcos del Jalon 118, 28037 Madrid, Spain

**Keywords:** oxidative stress, inflammation, glaucoma, retina diseases, age-related macular degeneration, diabetic retinopathy

## Abstract

Most irreversible blindness observed with glaucoma and retina-related ocular diseases, including age-related macular degeneration and diabetic retinopathy, have their origin in the posterior segment of the eye, making their physiopathology both complex and interconnected. In addition to the age factor, these diseases share the same mechanism disorder based essentially on oxidative stress. In this context, the imbalance between the production of reactive oxygen species (ROS) mainly by mitochondria and their elimination by protective mechanisms leads to chronic inflammation. Oxidative stress and inflammation share a close pathophysiological process, appearing simultaneously and suggesting a relationship between both mechanisms. The biochemical end point of these two biological alarming systems is the release of different biomarkers that can be used in the diagnosis. Furthermore, oxidative stress, initiating in the vulnerable tissue of the posterior segment, is closely related to mitochondrial dysfunction, apoptosis, autophagy dysfunction, and inflammation, which are involved in each disease progression. In this review, we have analyzed (1) the oxidative stress and inflammatory processes in the back of the eye, (2) the importance of biomarkers, detected in systemic or ocular fluids, for the diagnosis of eye diseases based on recent studies, and (3) the treatment of posterior ocular diseases, based on long-term clinical studies.

## 1. Introduction

Age-related ocular diseases related to the posterior segment of the eye including glaucoma, age-related macular degeneration (AMD), and diabetic retinopathy (DR) share similar characteristics, which facilitates their diagnosis. However, all of them are serious diseases, leading to irreversible blindness. For instance, glaucoma is currently the most common cause of irreversible visual impairment. Additionally, different estimations predict the continuous increase of glaucoma in the coming years [1,2,3]. Moreover, the three diseases present a complex pathophysiology, which is related to cellular senescence, oxidative stress, and the inflammatory pathway [4].

Oxidative stress is normally associated with the generation of reactive oxygen (ROS) and reactive nitrogen species (RNS). ROS can react rapidly with nitric oxide (NO), generating RNS. These substances are considered as metabolites with a high capacity to oxidize proteins, lipids, and nucleic acids [5] and enhance autophagy and mitophagy processes [6], cell dysfunction, necrosis, apoptosis, and cell death [7,8].

### 1.1. Oxidative Stress and Inflammation

There are two main routes of ROS generation. The first is related to mitochondria, which are associated with the electronic transport chain [9,10] and to cytochrome P450 [11]. The second is related to nicotinamide adenine dinucleotide phosphate oxidase (NADPH oxidases), especially to phagocytic immune cells and endothelial cells [12], which are consequently associated with the inflammatory response [13].

Although the study of these substances focuses on the cellular damage products, they have important cellular functions such as regulators and signaling agents in multiple processes such as apoptosis, mitophagy, adhesion, and cell differentiation [6,14,15]. However, when the production of ROS and RNS exceeds the limits of the detoxification system in a prolonged or chronic way, these substances are considered the main mediators of the inflammatory pathology [5,13]. The pro-inflammatory activity of these substances is partially related to immune system cells such as polymorphonuclear neutrophils. These cells are very abundant at inflammation sites, where specific enzymes such as myeloperoxidase are involved in the transformation of ROS and the immune response [14]. Oxidative stress and inflammation share close pathophysiological processes, appearing simultaneously in many pathologies and suggesting a relationship between both phenomena [15]. 

Currently, the consensus describes the role of oxidative stress as one of the first events in the inflammation cascade [16,17,18]; however, the mechanisms by which these oxidizing substances are able to initiate and modulate inflammation are still unknown [5]. The retina is a tissue that is especially sensitive to oxidative stress with a high metabolic rate and oxygen consumption. The presence of photoreceptors that are rich in fatty acids makes the retina susceptible to oxidation [19]. In the same way, different pathologies that affect this tissue are closely related to inflammatory processes [20,21,22]. Therefore, many pathologies related to the posterior pole of the eye have been associated with these two processes, sharing their pathophysiology not only in oxidative stress but also in inflammation phenomena. Thus, ocular biomarkers associated with oxidative stress and inflammation represent a strategy in the diagnosis and monitoring of ocular diseases [4,23]. 

**Figure 1:**
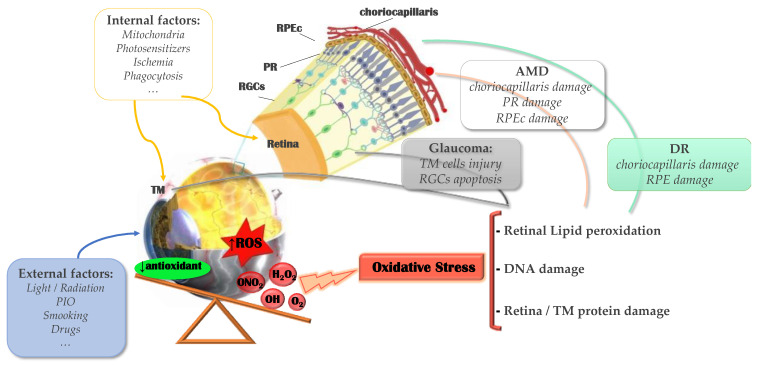
Scheme of oxidative stress role in posterior ocular diseases. IOP: intraocular pressure; ROS: reactive oxygen species; MT: trabecular meshwork; RGCs: retinal ganglion cells; PR: photoreceptors (cones + rods); RPE: retinal pigment epithelium; AMD: age-related macular degeneration; DR: diabetic retinopathy.

Before the classification of biomarkers, it is important to know their terminology. In general, a biomarker is defined as a measurable indicator of a relevant biological, clinical state, or it is capable of predicting one [24]. Biomarkers are typically molecules or structures easily obtainable from different parts of the body, fluids, or products, that can affect or predict the incidence of a disease [25]. They can be defined as molecular signatures of ocular diseases states and are detected in the major eye-derived fluids, including tears, aqueous humor, and vitreous humor, which may reveal critical information about the state of eye health. Their application is generally less invasive, faster and easier than the study of the final clinical state, and they are usually used for diagnosis and monitoring of the progress and prognosis of a disease [24]. In this review, we will focus specifically on biomarkers related to oxidative stress and inflammation in retinal diseases (including AMD and DR) and in glaucoma; see Figure 1.

### 1.2. Biomarkers of Oxidative Stress

The processes associated with oxidative stress present high concentrations of ROS and RNS. They comprise different chemical species such as superoxide anion oxygen (O_2_^−^), hydrogen peroxide (H_2_O_2_), and hydroxyl radicals (OH). RNS consists of nitric oxide (NO) and peroxynitrite (ONOO^−^), which results from the reaction of NO with O_2_^−^. [26]. Therefore, these chemical species have the potential to act as biomarkers of oxidative stress. In the same way, the evaluation of ROS generators such as enzymes, including nicotinamide adenine dinucleotide phosphate oxidase (NADPH oxidase) [27], show the same potential as biomarkers. Interestingly, numerous pro-inflammatory cytokines can activate NADPH oxidase and nitric oxide synthase 2 (NOS2), increasing NO production and consequently peroxynitrite [4].

Similarly, the mechanisms responsible for oxidative detoxification generate potential biomarkers. In this context, nuclear factor erythroid 2-related factor 2 (Nrf2) is a regulatory transcription factor for numerous detoxification enzymes through the sequence ARE (Antioxidant Response Element) [28,29]. Nrf2 has an impact when it comes to measuring the levels of glutathione (GSH), which is a tripeptide known for its active antioxidant role [30]. The synthesis of this tripeptide is catalyzed by glutathione peroxidase (GPx), and the GPx dysregulation is associated with processes of oxidative stress, inflammation, and upregulation of retinal vascular endothelial growth factor (VEGF) [31]. Endothelial changes involved in the activation of the vascular endothelium, related to inflammation and tissue regeneration processes, are promoted by VEGF [32].

### 1.3. Biomarkers of Inflammation

There are many biomarkers associated with inflammation, such as the matrix metallopeptidases (MMPs). In fact, MMPs are a family of calcium-dependent zinc-containing endopeptidases that is involved in the degradation of the extracellular matrix, apoptotic processes, and is closely related to inflammation [33,34]. In contrast, we find the transforming growth factor-beta (TGF-beta), which is an activator of extracellular matrix production with an anti-inflammatory role [35]. 

Another biomarker of inflammation is the tumor necrosis factor alpha (TNF-alpha). TNF-alpha is an intercellular signaling protein related to inflammatory processes and apoptosis; the regulation of this protein is related to the cytosolic concentrations of ROS [36]. TNF-alpha can stimulate the release of interleukin-6, which is a glycoprotein secreted by macrophages, T cells, endothelial cells, and fibroblasts involved in acute inflammation [37,38,39]. In addition, interleukins are a group of signaling cytokines closely related to the immune system [40]. The pro-inflammatory role is performed by other interleukins such as interleukin-1 and Interleukin-8, while others such as interleukin-10 have anti-inflammatory activity [39,41].

## 2. Oxidative Stress in Glaucoma

Glaucoma is a multifactorial optic neuropathy characterized by the damage of the optic nerve head and lamina cribosa, resulting in an irreversible loss of vision; this is the leading cause of irreversible blindness worldwide. It is estimated that about 76 million people will be affected by glaucoma worldwide in 2020 [1,42]. Glaucoma cause is still unclear, but it has been related to a mechanical stress and a reduction in the retinal blood that induces a gradual degradation of the retina, which is caused by the progressive damage of retinal ganglion cells (RGCs) and subsequently leads to their death [1]. There are several glaucoma subtypes, but the two most common ones are those that originate on the iridocorneal angle, including primary open-angle glaucoma (POAG) and primary close-angle glaucoma (PACG) [43]. 

Numerous risks or etiologic factors have been regarded as being involved in the pathophysiology development of glaucoma, such as increased intraocular pressure (IOP), aging, high glutamate levels, certain genetic susceptibility such as myocilin or optineurin alterations, alterations in NO metabolism, vascular alterations related with retina ischemia, and oxidative stress [44,45,46,47,48]. Nevertheless, the mechanism of RGCs death in glaucoma is not fully understood. However, it is known that RGCs are especially vulnerable to increased levels of oxidative stress due to their tremendous oxygen consumption and elevated proportion of polyunsaturated fatty acid. In this sense, there are two main theories used to explain the glaucoma physiopathology, mechanical and vascular theories, and, in both, the RGCs death is mediated by oxidative stress [49]. ROS, produced mainly by mitochondria, are signaling molecules that, in high levels, are able to activate apoptotic pathways, such as caspase 3 pathways (mitochondrial-mediated apoptosis related with cytochrome c release) [50] or caspase-independent pathways [51]. ROS levels reduction may protect RGCs from apoptosis [52], which are needed to maintain proliferation, signal transduction, and gene expression [53].

The most important is the mechanical theory that is based on increased IOP, which is currently considered to be the most important risk factor in developing glaucoma [47]. This IOP increase is generated by the incorrect balance between the production of aqueous humour via ciliary processes and the drainage of aqueous humour via the trabecular meshwork (TM) [53]. The mechanical theory highlights the importance of direct IOP-related increased compression of the axonal fibers, with deformation of the lamina cribosa plates and disruption of axoplasmic flow, resulting in the death of RGCs. In addition, the IOP increase induces changes in the mitochondria via its own fission, which stimulates reactive oxygen species production and is capable of accelerating oxidative adduct formation and increasing ROS-induced proteins such as heme oxygenase-1 (HO-1) [54,55]. This also causes abnormal cristae loss, cytochrome C production, and retrograde neurotrophic inhibition as well as a decrease in adenosine triphosphate (ATP) production [51,56,57], an enhancement of nitrite level and retinal lipid peroxidation, and a decrease in retinal antioxidants; furthermore, it stimulates glutaminergic neurotoxicity [58,59,60]. The imbalance between ROS and antioxidant agent concentration, in which ROS levels exceed the antioxidants concentrations, has been related with early retinal damage. 

At the same time, oxidative stress has been also implicated with trabecular meshwork, which is located in the sclerocorneal angle and bathed by aqueous humor. TM is the most vulnerable tissue of the anterior chamber to the oxidative damage due to its constant exposure to light, extremely active mitochondrial activity, and predisposition to inflammation. For that reason, TM contains antioxidant compounds to protect it from oxidative stress. The rise of oxidant–antioxidant imbalance decreases the protection of superoxide dismutase, catalase, and glutathione peroxidase, causing TM cell impairment mediated by ROS [58]. The TM degeneration is due to a cellular adhesion reduction to the adjacent extracellular matrix [61], overexpression of extracellular matrix proteins such as fibronectin, which reduces TM cell permeability [56,57], direct desoxyribonucleic acid DNA damage [59], and reduced local antioxidant activity [4,60].

Furthermore, it has been demonstrated that high levels of H_2_O_2_ are related to a resistance to the outflow of aqueous humor, which is presumably due to cytoskeleton rearrangements and the subsequent loss of adhesion of TM cells to extracellular matrix proteins, and TM cell loss induces an H_2_O_2_ effect [61]. In addition, the TM endothelium may release NO, which, in conjunction with free radicals, can worsen the metabolic conditions of TM cells and vary its motility [62,63]. NO acts as a cellular sodium pump modulator, encouraging glutamate production and other intercellular messengers and thus altering the activity of the ATP-dependent Na^+^/K^+^ pump, which produces a depolarization of the organelle, which is a mechanism implicated in glaucoma pathogenesis [64]. NO may also react with O- to form the potent neurotoxic peroxynitrite radical (ONOO-) in retinal neuron, this being more common in glaucoma models than control [65,66]. At the same time, anion superoxide radical (O_2_^−^), involved in biological membranes destruction, and hydroxyl radical (OH-), the most reactive free radical, tend to react with neighboring molecules such as DNA, lipids, or proteins, altering mainly the structure and fluidity of the TM cell membrane. In addition, DNA can be damaged by ROS, resulting in mutations that affect the non-cycling cell population locked in the G0 phase of cell cycle, which in TM has been reported as a potential pathogenetic factor for POAG onset [67]. The 8-hydroxy-2′-deoxyguanosine (8-OH-dG) is the most abundant oxidative nucleotide modification, and its concentration is significantly correlated with an increase in IOP and visual-field damage [68,69]. 

On the other hand, the RGCs death vascular theory focuses on compromised blood flow in retinal vessels, leading to an impaired autoregulation of blood flow to the optic nerve and the subsequent ischemia-induced production of ROS, such as hydroxyl radicals, which are the major cause of retinal injury [49,70]. The vascular dysfunction and neurodegeneration at the retina might be mediated by advanced glycation end products (AGEs), which are an oxidative stress-related biomarker that results from the reaction between reducing sugar with amino groups in proteins, lipids, or nucleic acids and is detected in the axons of RGCs and retinal glial cells in glaucoma. AGEs might activate signaling molecules as mitogen-activated protein kinases (MAPK) and nuclear factor kappa B (NF-kB), which induces ROS production, and the subsequent angiogenesis and neural apoptosis, which are related to immune responses as further described below [71]. In this way, Hondur et al. reported that AGEs were higher in aqueous humor and blood samples in glaucomatous patients than in non-glaucomatous healthy patients [60]. In addition, oxidative stress modifies retinal glutamate/glutamine cycling, leading to a rise of neurotoxic levels of glutamate, which induces a cellular components injury and is mediated by calcium, causing a depolarization of the organelle and an excessive ROS generation [58,72,73]. In this sense, it has been described that RGC apoptosis during glaucomatous injury itself generates ROS production; hence, excess ROS produce oxidative stress, which also harms the retina by causing a secondary degeneration of RGCs and generating a positive feedback cycle [72,74]. 

At the same time, there are common oxidative stress pathways linking vascular and mechanical theories. On one hand, blood flow decreases may be caused by the mechanical compression of the vessel walls, which is induced by a rise in IOP, affecting the blood supply to the laminar segments and damaging the RGC axons [49]. The rise in IOP induces a vascular dysregulation in the retina because of an excessive ROS production as well as an increase for NADPH oxidase 2 and lectin-type oxidized LDL receptor 1 (LOX1) expression and/or an endothelial dysfunction in retina arterioles [75,76]. On the other hand, vascular dysregulation might be related to TM damage, which is specifically linked to the production of oxidizing free radicals in TM performed by endogenous aerobic metabolism. In addition, the MT endothelium may release endothelins, which can induce vasoconstriction and subsequently TM motility, vessel perviousness, and IOP alterations [77]. However, the MT endothelium can also induce ischemia unrelated with vasoconstriction by a reduction of the activity of the ATP-dependent Na^+^/K^+^ pump [62,64]. 

Finally, there are also other pathogenic mechanisms related to oxidative stress-mediated glaucoma pathogenesis, such as inflammation activated by ROS and glutamate excitotoxicity, which are not related to a rise in IOP or vascular dysfunction [78]. An anomalous immune response and glial cell dysfunction may mediate oxidative stress, which harms RGCs indirectly [58,73]. In this sense, it has been described that apoptosis signal-regulating kinase 1 (ASK1) mediated apoptotic pathway acts by decreasing TNF-α signalling, which is a neurodegeneration mediator in glaucoma involved in the regulation of cytokine-induced apoptosis [79,80]. ASK-1 deletion has been shown to prevent RGC death in glaucoma animal models [79], and ASK1 deficiency has been linked to oxidative stress levels reduction and the subsequent RGC survival in glaucoma [81,82,83]. Moreover, an elevated ROS concentration induces NF-kB activation both in the retina and MT, which stimulates the expression of pro-inflammatory biomarkers, including endothelial leukocyte adhesion molecule-1 (ELAM-1), interleukin-1α (IL-1α), interleukin-6 (IL-6), and interleukin-8 (IL-8) [84]. 

## 3. Oxidative Stress in the Retina 

### 3.1. Age-Related Macular Degeneration 

Age-related macular degeneration (AMD) is the leading cause of permanent and irreversible blindness in patients over the age of fifty in developed countries [85]. It is a neurodegenerative disease that affects the central retina, called the macula, resulting in a progressive loss of vision [86]. The pathogenesis of AMD is complex and multifactorial, involving the interaction of genetic, metabolic, functional, and environmental factors. The major abnormalities take place in the ocular structures of the macular region presented in four interrelated tissues that include photoreceptors (PR), retinal pigment epithelium (RPE), brunch membrane (BM), and choriocapillaris [87]. Two forms of AMD are classically distinguished: the dry form, the most common, is characterized by the degeneration and death of photoreceptors and RPE cells [88]. The exudative form, the most rapidly progressing form, is linked to choroidal neovascularization (CNV) with angiogenesis bleeding and fluid leakage, leading to sudden loss of central vision [89]. The two forms share the same clinical features such as the presence of a lipid-like deposit called drusen in early AMD, modification in the pigmentation of RPE in the retina, and loss of vision due to geographic atrophy and neovascularization [90].

The retina is particularly susceptible to aging and vulnerable to the oxidative stress since its two components PR and RPE are highly metabolically active [91]. In the PR cells, there is a high demand for oxygen and nutrients from the blood cells and high metabolic activity. Thus, the retina is considered one of the highest oxygen-consuming tissues in the human body, making the retina oxygen tension over 70 mmHg [92]. This favorable environment, with abundant photosensitizers, visible light exposure, and a high energy demand, supports a highly oxidative milieu [91].

Furthermore, under normal conditions, RPE participates in the visual cycle, phagocytic uptake, and degradation of shed apical photoreceptor outer segments (POS) [93]. However, in the early stages of the disease, a crucial event in the molecular pathway is described by a drastic reduction of RPE cell functions. In fact, RPE progressively degenerate, leading to the degeneration of PR. Age-dependent phagocytic and metabolic insufficiency of RPE cells leads to a dysfunction of RPE and the progressive accumulation of lipofuscin granules [87]. Moreover, exposure to visible and ultraviolet A (UVA) light and high oxygen levels as described before in the eye cause oxidation reactions and modify the composition of lipofuscin. Consequently, the dysregulated lipid metabolism promotes the oxidative process in the retina. Other photoreactive molecules with lipofuscin are a potent photoinducible generator of reactive oxygen species (ROS), causing damage to both proteins and lipids [87]. In this stress environment, the photooxidation of lipofuscin generates reactive photoproducts including N-retinylidene-N-retinylethanolamine (A2E), DNA oxidation, and cells apoptosis [94].

Oxidation levels increase in the aging macula, even though the retina and RPE cells are rich in antioxidants such as vitamins (A, C and E) and carotenoids. As a result, augmented levels of ROS with an attenuated antioxidant cell defense system lead to oxidative stress, causing a critical site of injury in AMD characterized by more damage of PR, RPE cells, and choriocapillaris [95,96]. As previously mentioned, the retina has a very high oxygen consumption, and consequently, the stimulated retina tissue is abundant in ROS. Moreover, the phagocytosis of the photoreceptors outer segment (POS) led by RPE cells is accompanied by respiratory burst and rapid eruption of ROS [97]. Then, the digestion of POS induces the formation of more superoxide anion [98]. Furthermore, with the high-energy light exposition, polyunsaturated fatty acids (PUFA) present in the cell membranes of photoreceptors are readily oxidized. Gradually, peroxides and organic radicals progressively develop, with the oxidation of PUFAs accumulating in photoreceptors. In addition to this, the oxidation of PUFAs lasts many years and leads to the functional and structural impairment of cell membranes that leads to the degeneration of photoreceptors [99].

Additionally, PR and RPE, which are highly metabolically active, are composed of postmitotic cells. They particularly accumulate DNA mitochondrial damage resulting from their inability to reduce defective mitochondria during mitosis [92]. In addition to that, oxidative stress causes mitochondria impairment in aging RPE cells, with its changes in number, size, matrix density, and membrane integrity. This process is accompanied by mitochondrial mutations [100]. Chronic increases in oxygen radical production in the mitochondria can lead to a catastrophic cycle of mitochondrial DNA (mtDNA) damage as well as functional decline, further oxygen radical generation, and cellular injury [101]. However, these mitochondrial dysfunctions lead to low ATP levels, causing not only the attenuation of mitochondrial membrane potential but also the reduction of cytoplasmic calcium accompanied by the augmentation of mitochondrial calcium sequestration. Other damage includes chronic mitochondrial oxidative stress, leading to a decreased level of mitochondrial superoxide dismutase and consequently an increase in superoxide anion, shortening and disorganization of the photoreceptors, degeneration of RPE cells, thickening of Brunch’s membrane, and finally apoptotic cell death in the AMD process [102].

However, due to oxidative stress, there is a decline in the upregulation of autophagy in AMD. Nrf2 is the master regulator of the cellular antioxidant mechanism. In fact, it is a transcription factor that regulates the production of antioxidant enzymes against oxidative stress. Under normal conditions, Nrf2 is bound to Kelch-like epichlorohydrin (ECH)-associated protein 1 (Keap1) in the cytosol, inactive, and predestined for degradation by the ubiquitin–proteasome pathway [103]. However, under oxidative stress, Nrf2 dissociates from Keap1, resulting in its upregulation and translocation into the nucleus. This leads to the upregulation of several antioxidant genes and enzymes against ROS, including heme oxygenase 1 (HO-1), NAD(P)H-quinone oxidoreductase (NQO1), glutathione S-transferase (GST), superoxide dismutase (SOD), glutathione reductase, and ferritin [104]. Here, oxidative stress leads to the increase of different organic radicals and more ROS. For example, the O_2_^−^ radical is a highly potent oxidative agent, as each free radical rapidly gains three electrons to rebalance itself. Consequently, other ROS are generated, particularly hydrogen peroxide and hydroxyl radicals [86].

Finally, oxidative stress leads to chronic inflammation in the AMD process. In fact, the products of the oxidative stress trigger a chronic low-grade inflammation process. ROS impair cells’ functions, react with nucleic acids, proteins, and lipids, and induce the production of pro-inflammatory cytokines and angiogenic signals, including the development of new fragile blood vessels with the production of vascular endothelium growth factors (VEGF) [105] and changes in matrix metalloproteinases (MMPs) [106]. The inflammation process stimulated by the complement system and carried out in the Brunch membrane leads to different AMD forms. It is connected not only with the microglial activation in the retinal choroidal interface but also with autoantibodies and the formation of immune complexes in the Brunch membrane accompanied by choroidal macrophages infiltration, leading to CNV. During inflammation, the increased metabolic activity of the inflamed retina leads to the increased consumption of oxygen and causes hypoxia in the retinal cells. Chronic retinal hypoxia can lead to cell death and irreversible visual impairment observed in the exudative form of AMD [107].

### 3.2. Diabetic Retinopathy

Diabetic retinopathy (DR) is one of the microvascular diabetes complications. In fact, one-third of people with diabetes have DR. It is the major cause of blindness disease in the middle-aged and elderly people and is described as a progressive neurodegeneration [108]. According to the presence or absence of retinal neovascularization, DR can be classified clinically into non-proliferative (NPDR) and proliferative (PDR) forms with or without macular oedema [109]. In DR, every cell is exposed to abnormally extracellular high glucose concentrations that target retina and nerve tissues. The reason is that DR is characterized by chronic hyperglycemia, causing altered cellular homeostasis in the retinal microvasculature and endothelial cells in the choroid [110]. In the early stages of the disease, apoptosis causes the reduction of endothelial cells, which is followed by the increased number of acellular-occluded capillaries causing both the increase of vascular permeability and an increase of capillary membrane thickening, and causing edema and hemorrhages. Unsealed capillaries leak plasma and erythrocytes into the surrounding retinal tissue and lead to capillaries’ occlusion of the growth factors (such as VEGF) and pathological angiogenesis [111].

As previously described in AMD, oxidative stress also has an impact on DR. However, in diabetes, in contrast with AMD, increased oxidants and reduced antioxidant systems are present, independent of age, and have different negative effects [112]. In addition to hyperglycemia, inducing endothelial cells damage, ROS are generated mainly in the mitochondria, thereby stimulating mitochondrial superoxide production. Nevertheless, it is important to note that the progression of diabetic retinopathy is connected to ROS and oxidative stress mainly due to the metabolic memory [113,114]. Increased oxidant generation in the mitochondria might damage mitochondrial DNA and proteins, since ROS compromise the function of the electron transport chain. This damage leads to the synthesis of increased amounts of superoxide even with normal levels of glucose. In that case, even after normalized glycemia, DR progresses [113,114]. 

Mitochondrial dysfunction in both type 1 and type 2 DR accelerates premature endothelial cell apoptosis in the local oxidative stress and sustained hyperglycemia. Consequently, damage in mt DNA at the regulatory region is higher in comparison to other mt DNA portions. To remedy this, the overexpression of enzyme 8-oxoguanine DNA glycosylase (OGG1) and thymine DNA glycosylase is the result of mitochondrial DNA repair. Their transcription and replication mechanisms including mitochondrial transcription factor A (TFAM) and polymerase gamma (POLG) are also compromised [115]. The oxidative DNA damage marker is 8-hydroxy-2′-deoxy-guanosine (8-OHdG) with an increased level in RPE and choroid [116]. In DR, the origin and alteration in biochemical pathways are described as a chain of successive events linked to each other, from cause to consequence, with a snowball effect worsening the state of damage and oxidative stress [117]. In fact, hyperglycemia stimulates the increased mitochondrial ROS levels that activate the poly-ADP-ribose polymerase (PARP) pathway. Superoxide causes an elevation in the levels of glyceraldehyde-3-phosphate (G3P) by inhibiting its adenine dinucleotide + (NAD+)-dependent conversion to 1,3-diphosphoglycerate via the inhibition of glyceraldehyde-3-phosphate dehydrogenase (GAPDH) activity [118]. This mechanism reduces glyceraldehyde-3-phosphate dehydrogenase (GAPDH) activity, contributing to the overactivation of four classic hyperglycemia-induced metabolite pathways: (1) the polyol pathway, (2) the protein kinase C (PKC) pathway, (3) the Advanced Glycation End products (AGEs) pathway, and (4) the hexosamine pathway. All these four metabolites resulting from different molecular pathway become the source of ROS production and stimulation of oxidative stress [119]. In this regard, G3P, in high levels, play an important role in all the different pathways: G3P upregulates the formation and deposition of AGEs by accelerating the addition of triose phosphates to methyl-glyoxal, which is the main AGE precursor. G3P also upregulates the PKC pathway by enhancing the conversion of dihydroxyacetone phosphate to diacylglycerol (DAG) [120]. In continuation, G3P upregulation increases the availability of fructose 6-phosphate (F6P), which in turn drives flux through the hexosamine pathway to the enhancement of glucosamine-6-phosphate and ultimately uridine diphosphate-N-acetylglucosamine (UDP-GlcNAc) levels. Finally, G3P upregulation enhances the flux through the polyol pathway by increasing the availability of glucose [121]. The origin of the four hyperglycemias-induced pathway metabolites comes from the NADPH level. In fact, decreased NADPH levels and increased NADPH oxidase (NOx) levels contribute to the regeneration of glutathione, which is described as a scavenger of ROS. This imbalance in the level of NADPH and NADPH oxidase leads to ROS accumulation and cell damage [120]. Moreover, glycation of the metabolite AGEs causes mitochondrial dysfunction, and vice versa, persistent mitochondrial DNA damage and respiratory chain protein glycation generate AGEs that stimulate ROS production. More ROS amplified AGEs formation [115,120].

Oxidative stress not only influences mitochondrial dysfunction and retinal vasculature but also exerts a neurodegenerative impact on the diabetic retina. In fact, ROS decreases the brain-derived neurotrophic factor (BDNF), which regulates axonal growth, synaptic activity, and neuronal survival. Consequently, the damage of the synaptic transmitter and the degradation of the neurotrophic factor cause neuronal cells apoptosis and visual impairment [122]. Furthermore, oxidative stress is related to inflammation. ROS stimulates inflammation and angiogenesis by a molecular pathomechanism and contributes to the development of microvascular lesions. In this context, the AGEs pathway increases cytosolic ROS level and activates NF-*κ*B mechanism. [53].

Consequently, ROS regulate the expression of pro-inflammatory proteins by activation of the pro-inflammatory NF-κB pathway, which leads to the production of tumor necrosis factor alpha (TNF- α) and the generation of inflammatory and angiogenic mediators such as interleukins (IL-6), interleukine8 (IL-8), cyclooxygenase 2 (COX-2), intercellular adhesion molecule 1 (ICAM-1), monocyte chemoattractant protein 1 (MCP-1), VEGF, and different inflammatory cytokines [123]. Moreover, ROS derived from the family of NADPH oxidase (NOx) enzymes may activate hypoxia-inducible factor-1 (HIF-1) pathways and participate in the development of proliferative diabetic retinopathy and angiogenesis [123]. In addition, oxidative stress contributes to the pathogenesis of both diabetic micro- and macrovascular complications at the molecular level by apoptosis, the activation of stress signaling pathways, transcriptional factors, as well as in the induction of molecular damage of proteins, DNA, and lipids, accelerated formation of AGEs, and activation of homeostatic pathways [124,125]. Table 1 summarized the principal biomarkers of oxidative stress and inflammation in the back surface diseases.

## 4. Diagnosis

Current research lines on the diagnosis of eye diseases are focused on the detection of specific biomarkers in systemic or ocular fluids due to their potential in clinical practice. The biomarkers are catalogued as invasive biomarkers if they are obtained from aqueous humor, vitreous, or retina samples and non-invasive biomarkers if they can be obtained from urine, plasma, or tear samples. The differences between studies such as different analytical assays for detecting biomarkers or the variability in stages of the disease of the enrolled patients make it difficult to compare the results.

In this section, biomarkers of oxidative stress and inflammation process associated with glaucoma and retinal diseases are described.

### 4.1. Glaucoma

Advanced glycation end products (AGEs) could help in the early diagnosis and prognosis of glaucoma. Hondur et al. [60] reported significantly higher levels of AGEs in blood and aqueous humor (AH) samples in glaucomatous patients compared to control. AGEs accumulation can also be detected using a sensor that determines the skin autofluorescence levels, which is correlated with AGEs [71].

Products of oxidative stress are suggested as potential biomarkers. Nitric oxide (NO) is a free radical and shows higher serum [128] and aqueous humor concentration in patients with primary open angle glaucoma (POAG) [126,127]. Protein carbonyls (PC) content is the most general indicator and by far the most used marker of protein oxidation. An increase in levels is found in the serum of patients with pseudoexfoliative glaucoma (*PXG*) [128] and in aqueous humor of glaucomatous patients [60]. Malondialdehyde (MDA) is a lipid peroxidation product whose levels increase in plasma [128,129,130,131] and aqueous humor [137,155,190,191] in patients with glaucoma. Malonyl dialdehyde levels seem to be correlated with the severity of visual field loss in primary angle-closure glaucoma (PACG) and POAG [182,183]; thereby, they are considered to be a good indicator of the glaucoma progression. 8-hydroxy-2′-deoxyguanosine (8-OHdG) is an oxidative product of DNA damage whose levels in serum [67,132,133,134,135] and aqueous humor [67,134] are reported to be higher in glaucoma patients than controls. Mohanty et al. [134] found a strong positive correlation between plasma and aqueous 8-OHdG levels. Conversely, Kondkar et al. [135] concluded that 8-OHdG cannot serve as a potential clinical biomarker in POAG due to the high rate of false positivity measured with an ELISA kit. This agent can be also detected in urine but with lower correlation [133]. In general, the increase in oxidant agents is associated with a decrease of the antioxidant capacity. In this way, the fact that antioxidants such as superoxide dismutase (SOD) and glutathione synthase (GS) concentrations were significantly lower in POAG patients than in controls means that they may be used as biomarkers.

The inflammatory process results in the release of inflammatory cytokines and chemokines. In tear samples, an increase in IL-4, IL-12, IL-15 [192], IL-6 [136], and IL-8 [137] in patients with glaucoma have been reported. Agarkov et al. [138] proposed IL-2, IL-17, and IL-8 as good markers in tear film for use in the diagnosis and prognosis of glaucoma. Since the extraction of aqueous humor samples from patients is an invasive procedure, tear samples represent a non-invasive method that has attracted clinical interest. For this reason, the correlation between tears and aqueous humor has been evaluated but, until now, poor levels of correlation have been observed [184,192].

In aqueous humor, IL-5, IL-12, IL-15, IFN-γ, macrophage inflammatory protein-1 alpha (MIP-1 α) [192], macrophage inflammatory protein-1 beta (MIP-1β) [139], (IL)-8 [147,151,193], monocyte chemoattractant protein 1 (MCP-1) [139], and interferon gamma-induced protein (IP)-10 (24) were significantly higher in patients with a form of glaucoma. Chono et al. [185] identified the highest odds ratio for IL-8 in PXG or neovascular glaucoma (NVG), and its level was correlated with preoperative IOP or visual field defects in PXG eyes. They showed that the level of IL-8 in the aqueous might be a potential candidate molecule that can predict the clinical outcome of surgical interventions in eyes with refractory glaucoma. Elevated levels of tumor necrosis factor alpha (TNF-α) can induce retinal ganglion cell (RGC) apoptosis in patients with glaucoma, and for this reason, its expression has been studied [194]. High levels of TNF-α in plasma [140,141] and aqueous humor [142,143,144] have been associated with POAG and PXG, and its potential as a biomarker for glaucoma diagnosis or progression has been suggested [142]. TNF-α can be utilized as a predictor of the outcomes of glaucoma surgery [195].

Vascular endothelial growth factor (VEGF) is a cytokine with a significant role in neovascularization and inflammation. High levels of VEGF in aqueous humor are increased in POAG [145,146] and have been mainly associated with NVG [147,153,154,196,197]. VEGF levels have been correlated with other cytokines [185], and it is postulated that its expression relates with the severity of glaucoma and plays a role in glaucoma development and progression in NVG.

In general, an increase in MMP-9 activity in AH and in tear samples of patients with POAG and in early forms of PACG, POAG, and *PXG* eyes compared to controls have been reported [147,148]. Concentrations of MMP-9 in the tear film have been employed in the development of a linear multivariate regression analysis for predicting the onset and progression of POAG [198].

### 4.2. Retinal Diseases

#### 4.2.1. Age-Related Macular Degeneration 

Inflammatory cytokines and chemokines are proposed as potential biomarkers. In aqueous humor, Sakurada et al. [156] found significantly higher levels of IL-1α, IL-15, IP-10, and C-reactive protein (CRP) in neovascular age-related macular degeneration (nAMD) patients. Whereas some studies showed an increase of IL-6 levels associated with the pathology [157,158], others did not find the statistical significance [190,191,199]. IL-6 has been associated with the presence of geographic atrophy secondary to AMD [200]. More aqueous cytokines associated with nAMD include VEGF [156,201], monocyte chemoattractant protein 1 (MCP-1) [159,160,161], migration-inducing gene (MIG) [159], and TGF-ß. In contrast, other studies did not report a significant difference in aqueous VEGF [164,167,202] and MCP-1 levels [162,163,164]. In vitreous, higher IL-1B levels and transforming growth factor-ß (TGF-ß) were found in nAMD patients [165,166].

Regarding tear film, a pilot study performed by Winiarczyk et al. [167] found the upregulated expression of inflammatory markers such as myosin-13 and signal transducer and activator of transcription 3 (STAT3) in nAMD patients. STAT3 has been postulated to be a potential biomarker for the diagnosis of AMD. In dry AMD, there was a major representation of two proteins, calpain-7 (CAPN7) and Myc proto-oncogene protein (MYC), which are involved in oxidative stress and inflammation. MMP-9 has been found in the aqueous humor of nAMD patients [159]. In the vitreous of AMD patients with subretinal fluid (SRF) accumulation, the levels of MMP-9 showed a positive correlation, suggesting it as a prognostic biomarker for diseases affected by SRF accumulation [168].

Factors related to oxidative stress could be potential biomarkers for the incidence and/or progression of AMD. 8-hydroxy-2′-deoxyguanosine (8-OHdG) is an oxidative product of DNA damage whose levels are higher in the aqueous humor of nAMD patients [149,150]. The presence of malondialdehyde (MDA), one of the reactive compounds originating from PUFA oxidation, is detected in blood samples. Serum and plasma samples from AMD patients showed higher MDA levels than in the control groups [151,152,153,154,155]. They represent a reliable non-invasive biomarker of oxidative stress in AMD patients. Another lipid peroxidation marker is the F2-isoprostane (F2-IsoPs), which is considered to be important as a vivo marker of oxidative damage in AMD (63). Sabanayagam et al. [203] demonstrated that the presence in the urine of F2-IsoPs was positively associated with AMD.

#### 4.2.2. Diabetic Retinopathy

Some of the well-known AGE adducts described in vivo are pentosidine, N-(carboxymethyl) lysine (CML), and hydroimidazolone. Serum levels of pentosidine, hydroimidazolone, and CML increase in patients with type 2 diabetes retinopathy [178,204,205]. In aqueous humor, CML levels increase throughout the progression of diabetic retinopathy (DR) [169,170] and thus are used as markers of oxidation. In contrast, no correlation between AGE levels and retinopathy in diabetic patients has been found in some reports [193]. 

Regarding the inflammatory process, in tears, lactotransferrin, lipocalin 1 (LCN-1), lysozyme C, lipophilin A, lacritin, and immunoglobulin lambda chain levels increased in patients with diabetic retinopathy [173]. In contrast, Kim et al. [196] found a decreased of LCN-1 levels, along with heat shock protein (HSP) in no proliferative diabetic retinopathy compared with control. TNF-α levels increase in patients with DR and are correlated with the severity of the pathology [206,207]. In aqueous humor levels of IL-1β [175,197], IL-8, IP-10 [174], IL-6 [175,176,177,197], TNF-α [187,208,209], MCP-1 [174] (24), IL-2, IL-5 [175], and VEGF [169,174,176,177,178] were higher in patients with certain type of DR compared to controls. Most of the proteins reported in aqueous are correlated with vitreous: IL-6 [177,179,180,181], VEGF [182,183,184,185], IL-8 [184,192,194], IP-10 [186], MIP-1β, TNF-α [180], MCP-1 [184,192,210], PlGF [180], and VEGF. The vitreous and aqueous levels of IL-6 and VEGF were significantly correlated with the severity of diabetic retinopathy and with the pathogenesis of diabetic macular edema [183,189,211]. On the other hand, proteomics analysis of vitreous and AH shows proteins that participate in a complementary system: clusterin, complement C3, and C4-A, which are factors that can serve as biomarkers for proliferative diabetic retinopathy (PDR) [187]. 

Oxidative stress stimulates intercellular adhesion molecules (ICAM-1), vascular cell adhesion molecules (VCAM-1), and selectins (E-selectin), which mediate leukostasis—a typical event of the inflammatory process. The ICAM-1 level has been shown to be increased in the diabetic retina [171,172]. However, the results of these inflammatory markers are not consistent. While some studies reported differences in levels of ICAM-1, VCAM-1 in serum [212,213,214], or vitreous [215] and their possible relation with RD, others did not find significant differences [202,216,217]. Regarding matrix metallopeptidases, elevated concentrations have been reported in the retina of diabetic rats [218]. In vitreous [204] and in plasma samples, MMP-9 showed higher concentrations in DR [188] and PDR patients [189] than controls. 

## 5. Treatment

### 5.1. Glaucoma

The main therapeutical target for glaucoma treatment is decreasing the intraocular pressure by decreasing the aqueous humor production or increasing its outflow, as this pressure is the major risk factor for developing the disease [205]. Nevertheless, the use of antioxidants such as vitamins, coenzyme Q10, melatonin, essential fatty acids, and natural extracts, principally in the form of dietary supplements, has been studied and proposed as an adjuvant treatment.

The oral administration of nicotinamide (vitamin B3), a nicotinamide adenine dinucleotide (NAD+) precursor, demonstrated its efficacy in preserving the retinal ganglion cells in different rodent glaucoma models but also in improving the pattern electroretinogram [219,220,221], this last finding being confirmed in a randomized double-masked clinical trial in glaucoma patients [222]. Concerning ascorbic acid (vitamin C), Xu et al. [208] showed that its supplementation to a porcine trabecular meshwork culture correlated with both lower reactive oxygen species and higher lysosomal proteolysis, but in a clinical study by Leite et al. [209], the oral administration did not affect glaucomatous patients. Furthermore, the dietary deficiency of vitamin E was proved to be stimulating retinal ganglion cell death, which is associated with retinal lipid peroxidation, in a glaucoma rat model [210]. In this sense, commercial eye drops (Coqun^®^) combining vitamin E with coenzyme Q10, another antioxidant promoting the retinal ganglion cell survival and mitochondrial DNA preservation [211,223], were evaluated in glaucoma patients. These eye drops improved the pattern electroretinogram and reduced superoxide dismutase concentration in aqueous humor, but there was nothing reported about the main glaucoma clinical markers [224,225].

Melatonin is a neurohormone with antioxidant properties that has been studied for glaucoma treatment. However, its therapeutical interest lies in the agonist action on MT1, MT2, and putative MT3 melatonin receptors located in the ciliary body. The activation of these receptors decreases the chloride efflux from non-pigmented epithelial cells, reducing the aqueous humor production [226]. Through this mechanism, Martínez-Águila et al. [227,228] demonstrated that the topical instillation of melatonin and its analogs 5-methoxycarbonylamino N-acetyl tryptamine (5-MCA-NAT) and agomelatine reduced the intraocular pressure in rabbits and mice. In normotensive subjects, two clinical studies found that the short- and mid-term oral administration of nutritional supplements based on melatonin reduced by 1 mmHg the intraocular pressure [229,230], which are values not considered clinically relevant.

In relation to essential fatty acids, the oral administration of omega-3, omega-3 combined with omega-6, and α-lipoic acid in different animal glaucoma models showed efficacy in reducing retinal oxidative stress and inflammation, ganglion cell death, and even intraocular pressure [231,232,233,234]. Conversely, a single clinical study reported a decrease of only 1 mmHg in the intraocular pressure of normotensive subjects after the oral supplementation with omega-3 [235]. Additionally, the dietary supplementation of natural extracts based on Ginkgo biloba, green tea catechins, saffron, and black currant anthocyanins demonstrated antioxidant and neuroprotective properties in animal models [236,237,238] but no clinical efficacy to treat glaucoma [239,240,241,242,243].

Finally, the results of several clinical studies evaluating different dietary supplements combining antioxidants showed no influence on the main clinical glaucoma parameters or long-term development of the disease [244,245,246,247,248]. Therefore, this lack of scientific evidence makes more long-term studies vital to ascertain the need to incorporate antioxidant supplements for glaucoma treatment effectively.

### 5.2. Retinal Diseases

Similar to glaucoma, there are a large number of preclinical studies demonstrating the efficacy of vitamins B [249], C [250], and F [251], coenzyme Q10 [252], melatonin [253], omega-3 [254], α-lipoic acid [255], and different natural extracts [256,257,258] to protect the retina from oxidative stress. In different animal models of retinal degeneration and diabetic retinopathy, the antioxidant properties of these compounds have been associated with the inhibition of retinal cell apoptosis as well as reduced levels of both inflammation biomarkers and vascular endothelial growth factor (VEGF) [249,250,251,252,253,254,255,256,257,258].

Furthermore, other alternatives such as naturally occurring carotenoids, which are vitamin A precursors present in the retina [259], resveratrol [260], and synthetic drugs have been proposed as antioxidants treatments for retinal diseases. These synthetic drugs include free radical scavengers (edaravone and SUN N8075) [261,262], an antagonist of peroxisome proliferator-activated receptors (GSK0660) [263], an inhibitor of NADPH oxidase 1 and NADPH oxidase 4 (GKT137831) [264], and an activator of nuclear factor erythroid 2-related factor 2 (dimethyl fumarate) [265].

In the following sections, the efficacy of antioxidant dietary supplementation for age-related macular degeneration (AMD) and diabetic retinopathy treatment is reviewed, based on long-term clinical trials.

#### 5.2.1. Age-Related Macular Degeneration

The role of VEGF in the pathophysiology of AMD converted the anti-VEGF agents into the gold standard therapy of the disease, especially in its wet form [266]. Nevertheless, antioxidant supplements based on multivitamins, omega-3, trace elements, and natural extracts are usually prescribed as an adjuvant treatment for preventing and slowing the progression of AMD.

The Age-Related Eye Disease Study (AREDS), the most remarkable work in this context, was a multicenter, randomized, and double-masked clinical trial that involved 3640 AMD patients [267,268]. The study evaluated the long-term effects of the daily oral administration of a tablet containing vitamins C (500 mg) and E (400 IU), β-carotene (15 mg), zinc (80 mg), and cupric oxide (2 mg) on the AMD progression. In 2001, the 5-year follow-up results reported that the probability of progression to advanced AMD in patients who manifested high-risk clinical features was lower with AREDS formulation (20.2%) than the placebo administration (27.8%), manifesting an odds ratio (99% confidence interval) of 0.66 (0.47, 0.91) [267]. Later, the 10-year follow-up results showed that this probability increased to 45.7% in the group receiving the AREDS formulation, which was still lower than the placebo administration (53.8%), while the odds ratio remained at 0.66 (0.53, 0.83) [268].

The AREDS2 was a second clinical trial under the same experimental design that involved 4203 AMD patients with the purpose of assessing if the addition of lutein (10 mg) + zeaxanthin (2 mg), omega-3 (docosahexaenoic acid (350 mg) + eicosapentaenoic acid (650 mg)), or both to the original AREDS formulation would reduce the risk of AMD progression [269,270]. However, all of the formulations, including the original AREDS one, showed no effect on the risk of AMD progression to advanced stages compared with placebo administration [269]. Additionally, a higher incidence of lung cancer was found in AMD patients who received β-carotene compared with those who did not, especially in former smokers. The association between β-carotene and lung cancer was the reason why β-carotene was replaced by lutein and zeaxanthin in the commercially available AREDS formulation [270].

The Nutritional AMD Treatment 2 (NAT2) study was another important clinical trial that involved 263 exudative AMD patients for a 3-year follow-up, where the daily oral administration of docosahexaenoic acid (840 mg) and eicosapentaenoic acid (270 mg) only showed a lower incidence of choroidal neovascularization compared with placebo but with no statistically significant differences [271].

In two meta-analyses, Evans and Lawrenson [272,273] analyzed the capability of antioxidant dietary supplementation for both preventing AMD (five clinical trials) and slowing the progression of the disease (14 clinical trials), showing no evidence of the efficacy of multivitamin and other antioxidant supplements for being prescribed as AMD adjuvant treatments. Finally, other antioxidant drugs and natural extracts have been evaluated in clinical trials with limited efficacy to be incorporated in the clinical practice, too [274,275,276].

#### 5.2.2. Diabetic Retinopathy

Strict glycemic control is the first-line treatment of diabetes to prevent the ocular manifestations of diabetic retinopathy [277], but the dietary supplementation of antioxidants has been proposed as an adjuvant therapy with the same purposes as for AMD.

In this regard, several mid- and long-term prospective, randomized, and placebo-controlled clinical trials showed that the daily oral administration of tablets combining vitamins (A, B2, B3, B6, B9, B12, C, and E), carotenoids (lutein, zeaxanthin, and astaxanthin), coenzyme Q10, omega-3 (docosahexaenoic and eicosapentaenoic acids), and trace elements had a disparity of results in terms of blood levels of both antioxidants and glycated hemoglobin (HbA1c), visual function, or retinopathy signs in diabetes patients [278,279,280,281,282,283]. Out of the six referenced studies, only three studies reported an improvement in the blood levels of antioxidants [279,282,283], while one study reported an improvement in HbA1c [282], one study reported an improvement in visual function [280], and two studies reported an improvement in central macular thickness [279,282]. Additionally, García-Medina et al. [278], in a 5-year follow-up, found that the oral administration of vitamins C and E, lutein, β-carotene, and trace elements did not reduce the progression of diabetic retinopathy in patients with type 2 diabetes, this clinical trial being the only one that evaluated the efficacy to slow the progression of the retinal disease.

Concerning other dietary supplements, a clinical trial of Zhang et al. [284] reported that the daily administration of lutein 10 mg for 9 months in type 2 diabetes patients with non-proliferative diabetic retinopathy improved visual acuity in a clinically relevant way compared with placebo (0.10 LogMar). Conversely, Haritoglu et al. [285] did not report changes in visual function, in addition to blood levels of HbA1c and macular edema prevention after the daily supplementation of α-lipoic acid in patients manifesting mild to moderate diabetic retinopathy. Moreover, the effect of different natural extracts in diabetic retinopathy has also been investigated but showed no changes in the severity of the disease [286,287,288,289].

Again, the lack of long-term studies evaluating these antioxidant therapies with positive results in the progression of diabetic retinopathy makes it impossible to prescribe dietary supplements as adjuvants with an evidence-based guarantees. Finally, Table 2 summarizes the principal long-term clinical studies evaluating the effect of different antioxidant supplements not only in diabetic retinopathy but also in glaucoma and AMD.

## 6. Discussion

This review analyzed the oxidative stress and inflammatory processes in the back of the eye. The approach was to describe the role of oxidative stress as one of the first events in the inflammation cascade, thereby explaining how biomarkers of oxidative stress and inflammation are necessary to understand the physiopathology of main diseases and molecular crosstalk disorder that connect retinal blood vessels, retina, and retinal ganglion cells. For instance, in glaucoma, the TM degeneration induced by oxidative injury might cause a disorder in the aqueous humor outflow pathway and the subsequent intraocular pressure elevation [58], the oxidative stress in this case being a secondary factor in the mechanical theory of glaucoma pathogenesis.

In the retina, oxidative stress appears to be central in the development of AMD and is identified as a crucial factor in the progression of the pathology [290]. This is due to its relationship with other molecular mechanisms and physiological conditions that favor the generation of ROS and lead to dysregulated lipid metabolism, dysregulated antioxidant mechanisms, mitochondrial dysfunction, dysregulated angiogenesis, and inflammation. Moreover, in diabetic retinopathy, hyperglycemia seems to be the first trigger in the pathogenesis of vascular complications, and oxidative stress represents the common link in all of the hyperglycemia-induced biochemical and molecular pathways in the retina. Many metabolic and hemodynamic pathways and their relatives’ mediators are activated. Based on the strong evidence of a role of oxidative stress in the pathogenesis of vascular complications, the use of antioxidants should represent an appealing approach.

The treatment of these pathologies with antioxidant supplements is controversial. Several studies have shown that antioxidant may help to regulate the oxidative stress damage in the posterior pole of the eye in animal model, but it is not possible to extrapolate to our clinical practice, because human trials have not clearly shown the efficacy. In many cases, different antioxidants are combined seeking to improve the effect. This combination shows some disadvantages such as positive or negative interaction of different antioxidants among them with uncertain effects or the optimal dose and combination. It is evident that more studies have to be developed to evaluate the long-term efficacy and safety of different combinations of antioxidants in order to find out useful formulation against a degenerative posterior pole eye disease.

In summary, there is a clear importance of oxidative stress in posterior pole pathologies, but biomarkers of the inflammation related with oxidative stress, detected in systemic or ocular fluids, could be very important for diagnosis and even treatment with different antioxidant supplements.

## Figures and Tables

**Table 1:** Summary of the principal biomarkers of oxidative stress and inflammation in clinical studies evaluating their presence not only in glaucoma and diabetic retinopathy but also AMD.

Disease	Molecular Disorder	Biomarker	Sample Type	References
Glaucoma	Oxidative stress	AGEs	Blood AH	Hondur et al. [60]
		NO	Serum AH	Zanón-Moreno et al. [126,127]
		PC	Serum AH	Erdurmuş et al. [128] Hondur et al. [60]
		MDA	Plasma AH	Erdurmuş et al. [128] Rokicki et al. [128,129,130,131]
		8-OHdG	Serum AH	Sorkhabi et al. [67,132,133,134,135]
		SOD, GS	AH	Yuki et al. [133]
Glaucoma	Inflammation	IL-4, IL-12, IL-15 IL-6, IL-8	Tear	Benitez-Del-Castillo et al. [136] Duveshet al. [137]
		IL-2, IL-17, IL-8	Tear film	Agarkov et al. [138]
		IL-5, IL-12, IL-15 IFN- γ, (MIP-1β) IL-8, MCP-1(alpha) (IP)-10	AH	Mohanty et al. [134] Kokubun et al. [139]
		TNF-alpha	Plasma AH	Kondkar et al. [140,141] Sawada et al. [142,143,144]
		VEGF	AH	Tripathi et al. [145,146]
		MMP-9	AH Tear	Markiewicz et al. [147,148]
AMD	Oxidative stress	8-OHdG	AH	Lau et al. [149,150]
		MDA	Serum Plasma	Totan et al. [151,152,153,154,155]
AMD	Inflammation	IL-1α, IL-15, IP-10 CRP	AH	Sakurada et al. [156]
		IL-6	AH	Klein et al. [157,158]
		VEGF, MCP-1 MIG, TGF-ß.	AH	Jonas et al. [159,160,161]
		VEGF MCP-1	AH	Sakurada et al. [156] Mimura et al. [162,163,164]
		IL-1B, TGF-ß	Vitreous	Zhao et al. [165,166]
		Myosin-13 STAT3	Tear film	Winiarczyk M et al. [167]
		CAPN7 MYC	AH	Jonas et al. [159]
		MMP-9	AH	Jonas et al. [159].
		MMP-9	Vitreous	Ecker et al. [168].
DR	Oxidative stress	Pentosidinr, CML hydroimidazolone	Serum	Fosmark et al. [169,170]
		CML	AH	Endo et al. [169,170]
		ICAM-1	Retina	McLeod et al. [171,172]
DR	Inflammation	Lactotranferrine Lipophilin A, lacritin, Ig lambda	Tear	Csősz et al. [173].
		IL-1β IL-8, IP-10, IL-6 MCP-1 IL-2, IL-5 VEGF	AH	Oh et al. [174] Wu et al. [175] Endo et al. [169,174,176,177,178]
		IL-6, VEGF IL-8, IP-10 MIP-1β, TNF-α MCP-1, PlGF	AH	Funatsu et al. [177,179,180,181] Kakehashi et al. [182,183,184,185] Wu et al. [180,181,186] Elner et al. [186]
		Clusterin, complement C3, C4-A factor I	Vitreous AH	Balaiya et al. [187]
		MMP-9	Vitreous Plasma	Jacqueminet et al. [188] Beránek et al. [189]

**Table 2:** Summary of the principal long-term clinical studies evaluating the effect of different antioxidant supplements for the treatment of posterior ocular diseases.

Disease	Reference	Antioxidants	Study Design	Main Findings
Glaucoma	Parisi et al. [224]	Vitamin E, coenzyme Q10	Prospective (*n* = 43)	After the daily topical instillation of eye drops for 12 months, the electroretinogram pattern was improved in open-angle glaucoma patients with similar results to the monotherapy with β-blockers, manifesting no changes in the intraocular pressure.
Glaucoma	García-Medina et al. [244]	Vitamins A, B1, B2, B3, B6, B9, B12, C, E, lutein, zeaxanthin, omega-3, trace elements	Prospective, randomized (*n* = 117)	After the daily oral administration for 24 months, there were no changes in terms of visual field and retinal parameters evaluated by optical coherence tomography, in the macular and optic nerve, compared with the control patients.
Glaucoma	Mutolo et al. [245]	Vitamins B1, B2, B6, forskolin, homotaurine, carnosine, trace elements	Prospective, randomized, (*n* = 22)	After the daily oral administration for 12 months, the intraocular pressure decreased 1.9 mmHg, and the retinal function in terms of pattern electroretinogram and foveal sensitivity improved compared with the control group.
Age-related macular degeneration	Age-Related Eye Disease Study (AREDS) [267,268]	Vitamins C, E, β-carotene, zinc, cupric oxide	Prospective, multicenter, randomized, double-masked (*n* = 3640)	After the daily oral administration of the AREDS formulation for 120 months, the estimated probability of progression to advanced AMD in patients who manifested high-risk clinical features was lower with AREDS formulation (45.7%) than the placebo (53.8%), the odds ratio and its 99% confidence interval being 0.66 (0.53, 0.83).
Age-related macular degeneration	AREDS2 [269,270]	AREDS + (1) lutein and zeaxanthin (2) omega-3 (3) both together	Prospective, multicenter, randomized, double-masked (*n* = 4203)	After the daily oral administration of the AREDS2 formulations for 60 months, there was no risk of developing advanced AMD with any of the formulations, which included the original AREDS composition. Additionally, a higher incidence of lung cancer was found in a group receiving β-carotene vs. no β-carotene group, especially in former smokers.
Age-related macular degeneration	The Nutritional AMD Treatment 2 (NAT2) [271]	Docosahexaenoic acid, eicosapentaenoic acid (omega-3)	Prospective, randomized, double-masked (*n* = 263)	After the daily oral administration of the NAT2 formulation for 36 months, these antioxidants only showed a lower incidence of choroidal neovascularization compared with placebo but with no statistically significant differences.
Diabetic retinopathy	García-Medina et al. [278]	Vitamins C, E, lutein, β-carotene, trace elements	Prospective, randomized (*n* = 97)	After the daily oral administration for 60 months, patients with type 2 diabetes and non-proliferative retinopathy or without retinopathy showed a statistical reduction in the progression of retinopathy, which was not considered clinically relevant compared with the control group, and there were no changes in the plasma total antioxidant status.
Diabetic retinopathy	Lafuente et al. [282]	Vitamins A, B2, B3, B6, B9, B12, C, E, lutein, zeaxanthin, omega-3, trace elements	Prospective, randomized, single-blind (*n* = 55)	After the daily oral administration for 36 months, there was an improvement of visual function (not clinically relevant), central macular thickness, and the plasma levels of HbA1c, IL-6, docosahexaenoic acid, and other antioxidants.
Diabetic retinopathy	Sanz-González et al. [283]	Vitamins A, B2, B3, B6, B9, B12, C, E, lutein, zeaxanthin, omega-3, trace elements	Prospective, randomized (*n* = 480)	After the daily oral administration for 38 months, the blood levels of different pro-oxidants markers decreased, and the antioxidants increased in type 2 diabetic patients with diabetic retinopathy. No signs of ocular disease development were analyzed.

## Data Availability

Not applicable.
